# Whole-Genome Scanning for Selection Signatures Reveals Candidate Genes Associated with Growth and Tail Length in Sheep

**DOI:** 10.3390/ani14050687

**Published:** 2024-02-22

**Authors:** Taotao Li, Meilin Jin, Huihua Wang, Wentao Zhang, Zehu Yuan, Caihong Wei

**Affiliations:** 1 State Key Laboratory of Animal Biotech Breeding, Institute of Animal Sciences, Chinese Academy of Agricultural Sciences, Beijing 100193, China; ltt_ltt2020@163.com (T.L.); jmlingg@163.com (M.J.); wanghuihua@caas.cn (H.W.); m18251871965@163.com (W.Z.); 2 Joint International Research Laboratory of Agriculture and Agri-Product Safety of Ministry of Education, Yangzhou University, Yangzhou 225009, China; yuanzehu@yzu.edu.cn

**Keywords:** sheep, growth rate, body size, tail length, DCMS, candidate genes

## Abstract

**Simple Summary:**

Sheep are bred to provide livestock products for humans. At present, sheep production systems tend to be specialized and refined, and mutton sheep production is an important part of these systems. Compared to Chinese indigenous sheep breeds, Western sheep breeds have rapid growth rate, larger physique, and higher meat yield. Growth rate and body size are critical to mutton sheep production. The population structure and genome-wide selection signatures can be used to identify genetic differences in sheep growth rate and body size. Therefore, in this study, a genome-wide investigation of the growth rate and body size between Chinese indigenous sheep breeds and introduced sheep breeds was performed. Furthermore, we also investigated the tail length trait between long- and short-tailed sheep breeds. The results showed that we identified candidate genes associated with growth rate, body size, and tail type, and elucidated the potential function of these genes.

**Abstract:**

Compared to Chinese indigenous sheep, Western sheep have rapid growth rate, larger physique, and higher meat yield. These excellent Western sheep were introduced into China for crossbreeding to expedite the enhancement of production performance and mutton quality in local breeds. Here, we investigated population genetic structure and genome-wide selection signatures among the Chinese indigenous sheep and the introduced sheep based on whole-genome resequencing data. The PCA, N-J tree and ADMIXTURE results showed significant genetic difference between Chinese indigenous sheep and introduced sheep. The nucleotide diversity (π) and linkage disequilibrium (LD) decay results indicated that the genomic diversity of introduced breeds were lower. Then, F_st_ & π ratio, XP-EHH, and de-correlated composite of multiple signals (DCMS) methods were used to detect the selection signals. The results showed that we identified important candidate genes related to growth rate and body size in the introduced breeds. Selected genes with stronger selection signatures are associated with growth rate (*CRADD*), embryonic development (*BVES*, *LIN28B*, and *WNT11*), body size (*HMGA2*, *MSRB3*, and *PTCH1*), muscle development and fat metabolism (*MSTN*, *PDE3A*, *LGALS12*, *GGPS1*, and *SAR1B*), wool color (*ASIP*), and hair development (*KRT71*, *KRT74*, and *IRF2BP2*). Thus, these genes have the potential to serve as candidate genes for enhancing the growth traits of Chinese indigenous sheep. We also identified tail-length trait-related candidate genes (*HOXB13*, *LIN28A*, *PAX3*, and *VEGFA*) in Chinese long-tailed breeds. Among these genes, *HOXB13* is the main candidate gene for sheep tail length phenotype. *LIN28A*, *PAX3*, and *VEGFA* are related to embryonic development and angiogenesis, so these genes may be candidate genes for sheep tail type traits. This study will serve as a foundation for further genetic improvement of Chinese indigenous sheep and as a reference for studies related to growth and development of sheep.

## 1. Introduction

Sheep (*Ovis aries*) is one of the domesticated animals which has played an important role in diverse human societies as a source of food, hide, and wool. As production levels increase, more attention is paid to improving growth performance and mutton quality. Although China has rich sheep germplasm resources, its sheep production mode is still mainly small-scale scattered farming with family as a unit and Chinese indigenous sheep breeds exhibit slower growth rate and lower meat yields when compared to Western breeds [1]. In contrast, Western sheep productions have more specialized and intensive sheep industry system, and specialized breeds have been bred. So, these excellent Western breeds (such as Dorper, Texel, Hornless Poll Dorset, German Mutton Merino, and Australian Merino sheep) [2] were introduced to China for crossbreeding to rapidly improve the production performance and mutton quality of local sheep.

Growth rate is an important genomic trait for sheep producers because the live weight at slaughter or carcass weight determines the producers’ income [3]. Body size is genetically correlated with growth rate. High-throughput SNP genotyping has been used to perform GWASs in sheep to map growth rate and body size, and has discovered weight- and height-related genes, such as *NCAPG*, *LCORL*, and *HMGA2* [4], which appear to be shared across mammals for influencing body size. Compared to horses and dogs, there are more QTLs for body size in sheep, with smaller effect sizes [3,5]. The tail is a body size characteristic of chordates, which is formed by different numbers of tail vertebrae in different species and plays multiple functions, including its use as a fifth limb, as a visual signal for warning and courtship, and as an essential physiological and morphological driver for functions related to propulsion, stability and maneuverability, energy storage, and thermoregulation [6]. Different sheep breeds differ in tail length, directionality, and fat deposition. Although fat deposition mainly determines the tail-type phenotype, the length and number of the tail vertebrae are also considered to be important factors affecting tail morphology. The long-tailed phenotype and tail fat deposition of sheep in modern sheep farming have reduced economic efficiency and affected sheep production. *PDGFD*, *BMP2*, *HOXB13*, and *TBXT* have been reported to regulate tail phenotypes in sheep, including tail fat deposition and tail length [7,8,9,10,11]. Genome comparison between Chinese and Western sheep are relatively few, and there are few studies on the tail variation among Chinese long-fat-tailed breeds and short-fat-tailed breeds. At present, the development of omics technology is promoting the study of the genetic basis and internal regulatory mechanisms behind the phenotype of organisms. Thus, it is necessary to perform genome selection signature analyses to reveal candidate genes associated with sheep growth-related traits.

Many statistical tests have been used to detect selection signatures, such as F_st_, XP-EHH, and Tajima’s D. Due to the different information sources and statistical methods of single statistic tests, it often does not return consistent results. Prioritizing selection signals detected used several single statistical tests can overcome these issues to some extent and reduce false positive results, but some loci with weak selection signals were missed by using this strategy [12]. This prompted the generation of statistics based on combining *p*-values of different statistical tests (composite measures of selection) [13,14]. De-correlated composite of multiple signals (DCMS) is one of these composite statistics [14], which has a higher power than most of the single statistics and shows a reliable positional resolution [14]. This strategy has been used to scan yak and cattle selection signatures, but it has not been reported in sheep. It is necessary to search for putative genes among specific sheep populations using DCMS analysis. In this study, whole-genome resequencing data were used for the following analyses: (a) we analyzed the difference in population genetic structure between introduced breeds and Chinese indigenous breeds, (b) we identified the candidate genes associated with growth and body size in introduced breeds, (c) tail-length-related candidate genes in Chinese long-tailed breeds were also identified.

## 2. Materials and Methods

### 2.1. Sampling Information and Sequencing

All experimental procedures involving sheep were approved by the Animal Ethics Committee of the Institute of Animal Sciences, Chinese Academy of Agricultural Sciences (protocol code IAS 2020-82) (Beijing, China). A total of 54 1-year-old female individuals of 15 sheep breeds were used in this study, including 5 introduced breeds and 10 Chinese indigenous breeds. Individuals of the same breed were not related to one another. The sampling location of these breeds are listed in Supplementary Material (Table S1). The blood of each sheep was collected from the jugular vein for high-throughput resequencing. Genome DNA was extracted from blood using the phenol chloroform method. Sequencing libraries were generated using the Truseq Nano DNA HT Sample Preparation Kit (Illumina, San Diego, CA, USA). Whole genomes of the 54 sheep were sequenced on the Illumina Hiseq 2000 platform. Sequencing data were submitted to the National Center for Biotechnology Information database (BioProject ID: PRJNA521847). The samples’ information is shown in Table 1.

### 2.2. Quality Controlling, Reads Mapping, and SNP Calling and Annotating

The raw data of fastq format was first processed through a series of quality control procedures using FastQC (https://www.bioinformatics.babraham.ac.uk/projects/fastqc/ (accessed on 17 February 2024)) to ensure reliable reads in the next analyses. The standards of quality control were followed, including: (1) removing reads with ≥10% unidentified nucleotides (N); (2) removing reads with >20% bases having phred quality less than 5; (3) removing reads with >10 nt aligned to the adapter, allowing ≤10% mismatches; (4) removing putative PCR duplicates generated by PCR amplification in the library construction process (reads 1 and 2 of two paired-end reads that were completely identical).

The clean reads were aligned to the sheep reference genome (ARS-UI_Ramb_v2.0, https://www.ncbi.nlm.nih.gov/datasets/genome/GCF_016772045.1/ (accessed on 17 February 2024)) using BWA (https://sourceforge.net/projects/bio-bwa/files/ (accessed on 17 February 2024)) (v.0.7.17) (settings: mem -t 4 -k 32 -M) [15]. Duplicate reads were then removed using Samtools (v.1.15) (setting: rmdup) [16]. Next, we performed SNP calling using the Samtools (v.1.15) [17] and filtered the SNPs data to obtain high quality SNPs. The filtering procedures were as follows: (1) the coverage depth of SNP was more than 2, (2) the missing rate was less than 10%, (3) MAF (minimum allele frequency) >5%. The variants were annotated using ANNOVAR (https://annovar.openbioinformatics.org/en/latest/ (accessed on 17 February 2024)) based on the sheep annotation database from the National Center for Biotechnology Information database (https://www.ncbi.nlm.nih.gov/ (accessed on 17 February 2024)) [18].

### 2.3. Population Structure Analysis

Principal component analysis (PCA), phylogenetic tree, and population structure were performed to assess the population genetic structure. PCA was performed using GCTA (https://yanglab.westlake.edu.cn/software/gcta/ (accessed on 17 February 2024)) (v.1.24.2) [19] to discern genetic relationships among breeds, pairwise identity-by-state distances were calculated among all individuals using a total of 20,826,457 independent SNPs. And the first two components were plotted using with the R program ggplot package (https://www.r-project.org/ (accessed on 17 February 2024)). Phylogenetic trees can represent the evolutionary relationships among populations, in this study, a neighbor-joining (N-J) tree was constructed using MEGA7 (https://www.megasoftware.net/ (accessed on 17 February 2024)) [20]. ADMIXTURE (http://software.genetics.ucla.edu/admixture/download.html (accessed on 17 February 2024)) (v.1.3.0) [21] was used to investigate the ancestral composition of each individual with genome-wide unlinked sites, with 1 < K < 4, where K is the number of expected subpopulations in this study.

### 2.4. Nucleotide Diversity and Linkage Disequilibrium

Nucleotide diversity (π) was calculated between different sheep breeds using VCFtools (https://vcftools.sourceforge.net/ (accessed on 17 February 2024)), with a 50 kb window in every breed. Linkage disequilibrium (LD) was calculated based on the mean r^2^ values for pairwise SNPs using PopLDdecay software (https://github.com/BGI-shenzhen/PopLDdecay (accessed on 17 February 2024)). SNPs with a minor allele frequency >0.05 were used. The parameters of PopLDdecay were set to “-MaxDist 500”.

### 2.5. Selective Sweep Detection

Based on phenotypic differences, we performed selection signature analyses for two groups, including the difference of body size and growth rate between the introduced populations (TEX, AWD, DOP, AME, and GME with bigger body size and faster growth rate) and Chinese native populations (UJI, THF, STH, HUS, and SNT, these breeds have smaller body size and slower growth rate), and the tail length difference between long-tailed population (TAN, TON, LTH, GLT, and LLT) and short-tailed population (UJI, THF, STH, HUS, and SNT). Three methods were used for analyses based on the SNP data, including F_st_, π ratio, and XP-EHH. First, the F_st_ values were calculated using VCFtools (v.0.1.16), with 40 kb as the window and 20 kb as the step in the comparison of two groups. Then, VCFtools (v.0.1.16) was used to estimate the nucleotide diversity and Tajima’s D by a sliding window in which the window was 40 kb and the step size was 20 kb in every group. The logarithm of nucleotide diversity ratio (log_2_(π ratio)) was calculated using the R script, and the results of F_st_ and π ratio joint analysis were calculated to screen the hypothesized selected regions. The selected regions were the regions with both extremely high F_st_ value (top 5%) and π ratio value (top 5%).

For the XP-EHH analysis, we performed haplotype inference using Beagle (v.5.4), and XP-EHH values were calculated using the python scikit-allel package and performed based on a sliding window approach with 40 kb window. The regions with top 1% of XP-EHH values were identified as presumptive candidates.

### 2.6. DCMS Analysis

The four selection signal methods (F_st_, π, XP-EHH and Tajima’s D) were combined to calculate the DCMS scores. First, the statistical results of the four methods were converted to *p*-values based on fractional ranks (between 0 and 1) and analyzed with the appropriate one-tailed tests (F_st_ and XP-EHH with right-tailed test, and Tajima’s D and π with left-tailed test) using the *stat_to_pvalue* function of the R MINOTAUR package for all SNPs [22]. Next, the covariance matrix was calculated to obtain the correlations among these statistics using the *Cov-NAMcd* function (alpha = 0.75, nsamp = 300,000) of the rrcovNA R package. The matrix was then used as an input in the *DCMS* function of the MINOTAUR R package to calculate the DCMS values. The mean and variance of the DCMS values were estimated using the R MASS package *rlm* function to eliminate the influence of isolated values [23]. The outputs of the fitted model were used as the inputs in the *pnorm* R function to calculate the *p*-values of the DCMS statistics, and the DCMS *p*-values were then transformed into *q*-values (adjusted *p*-values) using multiple hypothesis testing from the R package *q*-value [24].

### 2.7. Variant Functional Annotations

Based on the reference assembly ARS-UI_Ramb_v2.0 genome, we used bedtools software (https://bedtools.readthedocs.io/en/latest/index.html (accessed on 17 February 2024)) to align the selected region to the reference genome annotation file to obtain candidate genes. For single selection signal method, the overlapped genes detected by three methods were identified as candidate genes. Then, the DCMS analysis results were annotated to obtain the selected genes with the *q*-value < 0.05. The functions of these candidate genes were further determined through the literature review.

## 3. Results

### 3.1. Sequencing and Variation Calling

In this study, whole-genome resequencing was performed on 54 individual sheep, and an average of 7.6× coverage data was generated. After aligning to the sheep reference genome (ARS-UI_Ramb_v2.0), a total of 9,581,315,830 reads were obtained, covering 98.03% of the reference sequence. A total of 20,826,457 SNPs were identified after variant calling and quality controlling. The SNP statistical results showed the variants mainly occurred in the intergenic, followed by the intron, and the exon regions. The exon variants included 75,476 non-synonymous SNPs and 63,547 synonymous SNPs (Table 2). This SNP dataset provides a new resource for sheep biological research and breeding.

### 3.2. Population Structure Analysis

**Figure 1:**
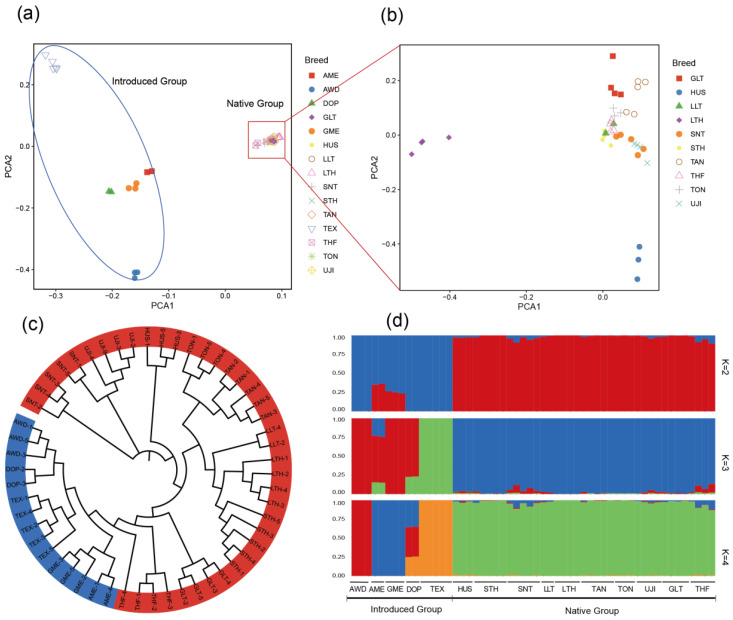
Population genetic structure of introduced breeds and Chinese native breeds: (**a**,**b**) principal component analysis (PCA) of all sheep individuals, and Chinese indigenous breeds, respectively. (**c**) neighbor-joining tree of 54 sheep. Introduced breeds are indicated in blue, and Chinese native breeds are indicated in red. (**d**) model-based clustering of sheep breeds using ADMIXTURE with K = 2 to K = 4.

Population genetic structure analysis was conducted based on the SNP data of 54 sheep. Firstly, the PCA results showed that all individuals could be grouped into two clusters, i.e., the introduced sheep population and the Chinese sheep population, suggesting a strong geographical distribution characteristic (Figure 1a). The Chinese indigenous breeds were further divided into two groups in the first principal component (PCA1), which were LTH, and the other 9 breeds (Figure 1b). Then, the phylogenetic tree (N-J tree) was constructed, and the result showed different clusters of 15 breeds, i.e., the 10 Chinese breeds in one clade, and the 5 introduced breeds (including TEX, AWD, DOP, AME, and GME) in the other clade (Figure 1c). Following this, the ADMIXTURE software (http://software.genetics.ucla.edu/admixture/download.html (accessed on 17 February 2024)) was used to estimate individual ancestries of all breeds. When K = 2, the CV-error value was the smallest, the optimal result was two subgroups, i.e., the introduced population and the Chinese indigenous population. It can be concluded that the 15 sheep breeds have 2 ancestral sources. When K = 3, TEX was separated from the other four Western breeds (Figure 1d). This was consistent with the results of the PCA and the N-J tree.

### 3.3. Nucleotide Diversity and Linkage Disequilibrium

**Figure 2:**
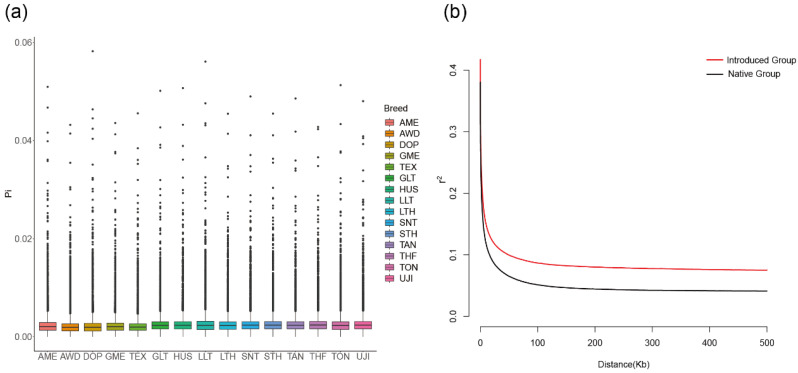
The nucleotide diversity of 15 sheep breeds (**a**) and the whole-genome average LD between the introduced population and native population (**b**).

The nucleotide diversity results showed that the Chinese native breeds had higher nucleotide diversity, while introduced breeds had lower nucleotide diversity (Figure 2a). From the perspective of linkage disequilibrium (Figure 2b), the introduced sheep population demonstrated a low decay rate and a high level of LD, whereas the Chinese native sheep population exhibited a high decay rate and a low level of LD. Overall, the LD result was consistent with the nucleotide diversity result, which indicated that the genetic polymorphism of the introduced population is lower.

### 3.4. Positive Selection Signatures in the Introduced Population

**Figure 3:**
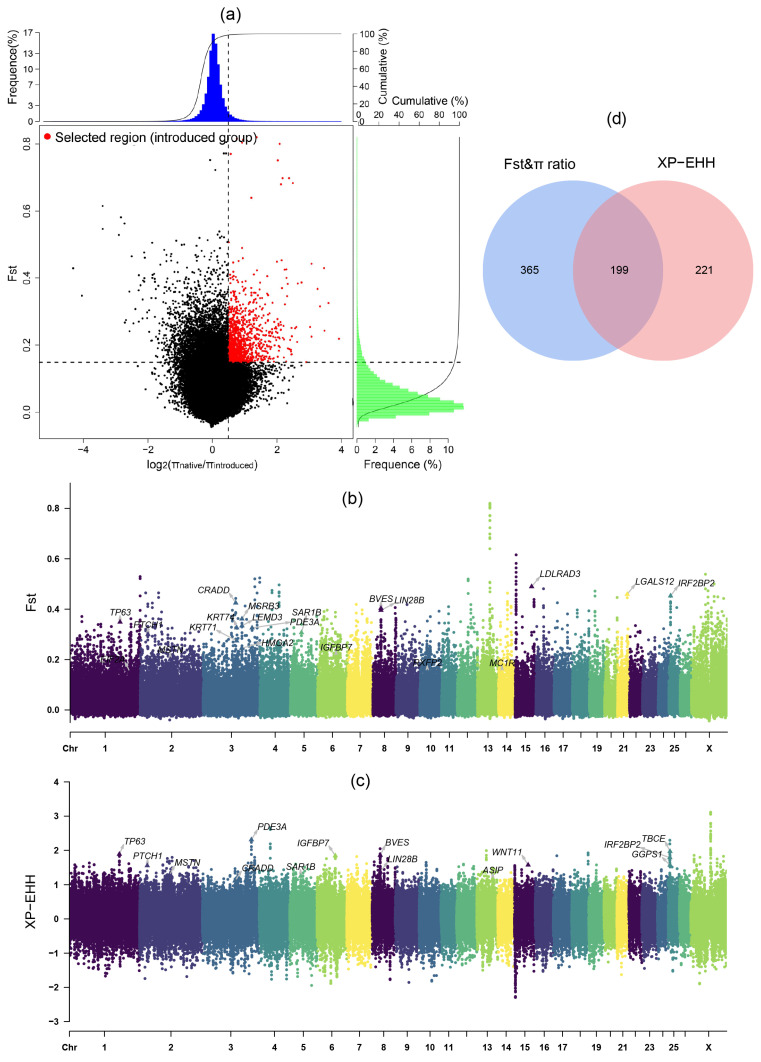
The results of selection signatures in the introduced sheep population. (**a**) The results of the combined analysis of F_st_ and π ratio. The selected regions for the introduced breeds are indicated by red dots. (**b**,**c**) Manhattan plots showing the selection regions in F_st_ and XP-EHH methods between introduced breeds and native breeds. (**d**) Venn diagram showing the overlapped genes from the F_st_, π ratio, and XP-EHH analyses.

**Figure 4:**
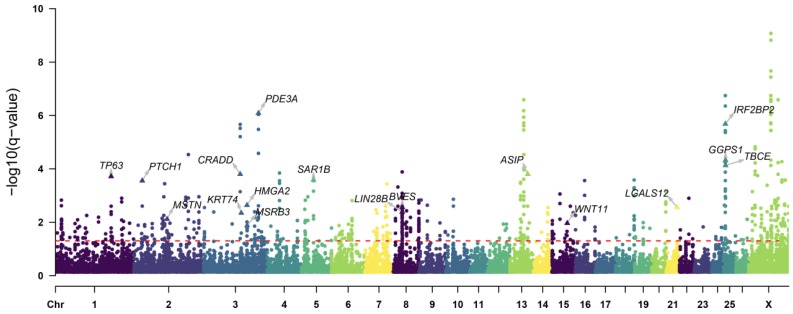
Manhattan plot showing the selected regions of the introduced breeds according to the DCMS method. The dotted line indicates the *q*-value = 0.05, and the data points above the dotted line are the selected regions.

To reliably detect genome variants related to the growth traits in the introduced population, we used the F_st_, π ratio, and XP-EHH statistics to scan the genome variations between the introduced population (TEX, AWD, DOP, AME, and GME) and the Chinese native population (UJI, THF, STH, HUS, and SNT). The F_st_ and π ratio analyses identified 564 genes (F_st_ > 0.15, log_2_(π_native_/π_introduced_) > 0.5) (Figure 3a,b), and the XP-EHH analysis identified 420 genes (XP-EHH > 1.05) (Figure 3c). We merged the gene lists generated by these three approaches and identified 199 specific genes that showed the strongest selection signatures (Figure 3d). Then, we also used one composite statistical method (DCMS) to scan the selection signatures. After the calculation of the DCMS statistics, the *p*-value was fitted to a normal distribution and corrected for multiple testing (FDR < 0.05). We identified 251 candidate genes with *q*-value < 0.05 (Figure 4, Table S2). Based on three single selection signal methods and one composite statistic (DCMS), we identified candidate genes with strongest selection signatures. These genes are associated with growth rate (*CRADD*), embryonic development (*BVES*, *LIN28B*, and *WNT11*), body size (*HMGA2*, *MSRB3*, and *PTCH1*), muscle development and fat metabolism (*MSTN*, *PDE3A*, *LGALS12*, *GGPS1*, and *SAR1B*), wool color (*ASIP*), and hair development (*KRT71*, *KRT74*, and *IRF2BP2*).

### 3.5. Positive Selection Signatures in the Long-Tailed Population

**Figure 5:**
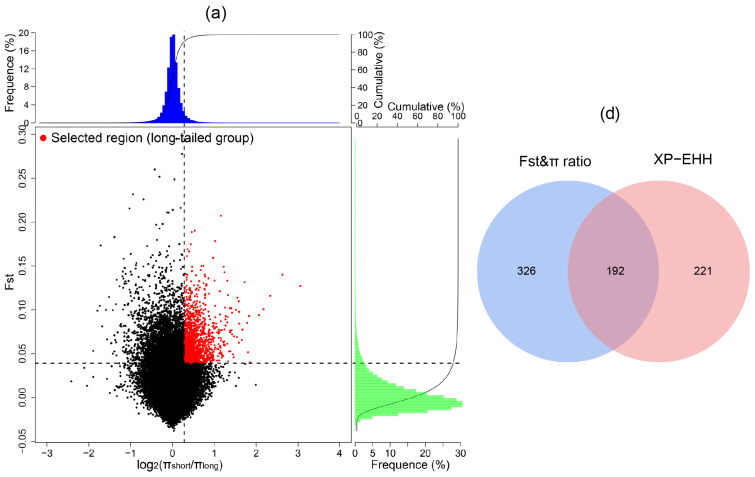

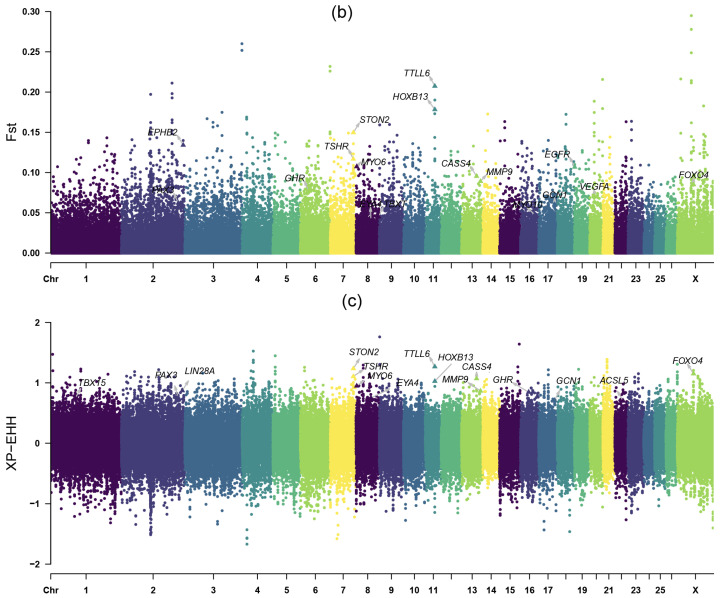
The results of selection signatures in long-fat tail sheep population. (**a**) The results of the combined analysis of F_st_ and π ratio. The selected regions for long-tailed breeds are denoted by red dots. (**b**,**c**) Manhattan plot showing the selected regions based on F_st_ and XP-EHH methods between the long-fat-tailed breeds and short-fat-tailed breeds. (**d**) Venn diagram showing the overlapped genes from the F_st_, π ratio, and XP-EHH analyses.

**Figure 6:**
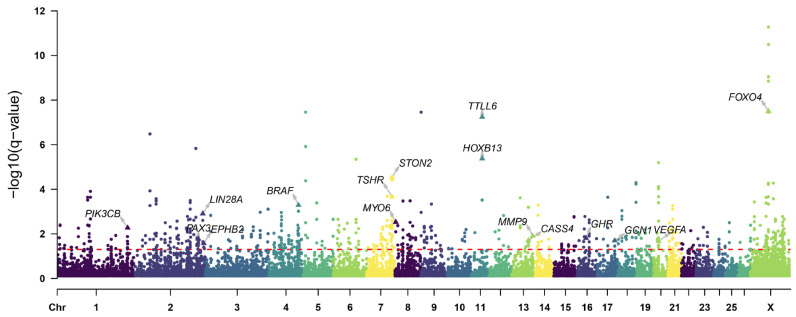
Manhattan plot showing the selected regions of long-tailed breeds according to the DCMS method. The dotted line indicates the *q*-value = 0.05, and data points above the dotted line are the selected regions.

Considering the variation in tail length among the Chinese indigenous sheep breeds, we classified these breeds into two groups based on their tail length: long-tailed breeds (TAN, TON, LTH, GLT, and LLT) and short-tailed breeds (UJI, THF, STH, HUS, and SNT), to identify selection signatures related to tail length phenotype. A total of 518 (F_st_ > 0.04, log_2_(π_short_/π_long_) > 0.28)) and 413 (XP-EHH > 0.7) genes were identified by F_st_ and π ratio and XP-EHH methods (Figure 5a–c), respectively, and 192 genes were identified as the overlapping genes of the three methods (Figure 5d). The DCMS analysis showed 246 candidate genes with *q*-value < 0.05 (Figure 6, Table S2). Among these genes, *HOXB13* showed the strongest selection signal with *q*-value < 4.22 × 10^−6^. Furthermore, we identified *LIN28A*, *PAX3*, and *VEGFA*, these genes regulate embryonic development and angiogenesis.

## 4. Discussion

Population structure and genetic diversity contribute to the evaluation of sheep genetic resources and play an important role in the utilization and conservation of genetic resources. After PCA, N-J tree and ADMIXTURE analyses, it was found that there were obvious differentiations between the introduced sheep breeds and the Chinese indigenous sheep breeds, the difference in geographical distribution leading to the distance in genetic relationship. After domestication, different sheep breeds were formed as they adapted to a diverse range of environments and specialized production systems during breeding and improvement [25]. For Chinese indigenous breeds, long-term natural selection has associated sheep traits with environmental adaptation, energy metabolism, and immune responses. Chinese indigenous sheep populations can be grouped into three lineages according to the explicit geographic patterns of tail type: Mongolian (fat-tailed sheep), Tibetan (thin-tailed sheep), and Kazak (fat-rumped sheep) [26,27]. The Chinese breeds in this study belong to the Mongolian lineage, the explicit geographic patterns were further evidenced by the distribution of genetic ancestries from fat-tailed sheep as inferred based on the population structure and PCA analyses, this further revealed a close genetic affinity among Chinese fat-tailed sheep breeds. However, under more highly specialized production systems of the Western sheep industry, sheep have formed specific phenotypic traits such as reproduction, presence of horns, and body size. In this study, Texel, Hornless Poll Dorset, Australian Merino, and German Merino sheep are breeds of a European origin, and Dorper is native to Africa. These breeds are cultivated for specific breeding purposes, and they showed great genetic divergence from the Chinese indigenous breeds. The nucleotide diversity and linkage disequilibrium decay results indicated a lower level of nucleotide diversity in the introduced breeds than the Chinese indigenous breeds, which was further evidenced by a higher degree of specialized breeding in the introduced breeds.

After scanning selection signatures, we obtained candidate genes that are associated with growth and development. For example, *CRADD*, *BVES*, *LIN28B*, *WNT11*, *HMGA2*, *MSRB3*, *PTCH1*, *MSTN*, *PDE3A*, *LGALS12*, *GGPS1*, *SAR1B*, *ASIP*, *KRT71*, *KRT74*, *IRF2BP2* identified in the introduced population are mainly associated with growth and body size, and *HOXB13*, *LIN28A*, *PAX3*, and *VEGFA* identified in the long-fat-tailed breeds are involved in tail development.

### 4.1. Selection Signatures for Growth Traits

In the face of the dramatic increase in mutton consumption, in addition to increasing the scale of sheep breeding, identification of candidate genes that regulate growth traits is being pursued, which not only meet demand for meat, but also effectively promote the development of Chinese sheep breeding.

Compared to the Chinese indigenous sheep population, the introduced sheep population are breeds for specialized production aims. They have a bigger body size and a faster growth rate. So, in our study, the selective sweep analyses identified potential candidate genes in the introduced population that may control the growth rate and body size in sheep. We found important selective scanning markers for growth traits in the introduced population, such as growth rate (*CRADD*), embryonic development (*BVES*, *LIN28B*, and *WNT11*), body size (*HMGA2*, *MSRB3*, and *PTCH1*), muscle development and fat metabolism (*MSTN*, *PDE3A*, *LGALS12*, *GGPS1*, and *SAR1B*), wool color (*ASIP*), and hair development (*KRT71*, *KRT74*, *IRF2BP2*). Among these genes, *CRADD* is associated with growth rate. *CRADD* encodes caspase-recruitment-domain and death domain containing protein [28], which is required for the activation of caspase-2-mediated apoptosis [29]. *CRADD* is expressed in many tissues and plays a role in regulating apoptosis in mammalian cells. *CRADD* has been identified as an excellent candidate gene for the high-growth phenotype in mice as its deletion produced 30–50% increase in weight gain of a systemic nature rather than tissue specifically [30,31]. *CRADD* was also related to body weight in Karachai goats at the age of eight months in a study by Ahmed et al. [32]. Chen et al. [33] found that the increase in copy number of *CRADD* by one in Luxi game chickens (a typical cockfighting breed) may be responsible for the fast muscle growth trait. Zhi et al. [34] identified that *CRADD* may be related to body size in Henan gamecock. Although, *CRADD* is not directly involved in growth-related pathways, it is involved in cell apoptosis, which is equally important in controlling cell number as cell proliferation is. An increase in cell numbers caused by the disruption of the apoptotic program caused by *CRADD* variation may result in a high-growth phenotype. In our study, *CRADD* was identified as a candidate gene in the introduced population, which may be associated with rapid growth.

Several embryonic-development-related genes were identified in this study, including *BVES*, *LIN28B*, and *WNT11*. *BVES* is essential for normal embryonic development [35], while *LIN28B* promotes cell differentiation and proliferation to ensure embryo elongation. *BVES* gene is highly expressed in cardiac and skeletal muscle cells, and can promote the regeneration of muscle cells through the cell adhesion function [36]. In a previous study, the mutation of *BVES* led to muscle dystrophy [37]. LIN28B is an RNA-binding protein that functions in development, maintenance of pluripotency and oncogenesis [38]. *LIN28B* has been reported to be associated with human height [39], especially the rapid height growth during puberty, indicating a critical role for *LIN28B* in the regulation of human growth [40,41]. *WNT11* plays a crucial role in the early development of vertebrate embryos [42], regulating tissue morphogenesis during gastrula formation. Interference with *WNT11* led to inhibition of axial elongation of the embryo on the anterior and posterior axes [43]. *WNT11* is also involved in the development of the skeletal system and contributes to bone homeostasis during osteogenic differentiation [44]. Alternatively, *MSRB3*, *HMGA2*, and *PTCH1* have plausible biological functions associated with body size. *MSRB3* encodes an antioxidant enzyme that catalyzes the reduction of methionine sulfoxide to methionine [45]. The *MSRB3* gene also contributes to ossification and fat deposition, and its mutation can cause the accumulation of oxidatively damaged proteins and reactive oxygen species (ROS), which in turn might affect normal development, morphogenesis and tissue homeostasis throughout body by activating caspases and initiating apoptosis [46]. Previous studies have reported that *MSRB3* influenced ear shape and size [7,47,48,49,50], the auditory system [46], and growth traits [32,51]. Therefore, the selection of this gene in the introduced population is associated with faster growth rate and relatively larger body size. *HMGA2* encodes an architectural transcription factor that regulates the transcription of a variety of genes and directs cellular growth, proliferation, and differentiation. Its mutation is associated with body size in dogs [52], horses [53], and pigs [54], and body height in horses [55] and cattle [56]. Therefore, it has been identified as one of the most important genes in regulating body size in vertebrates. *PTCH1* is another gene associated with body size. *PTCH1* is a tumor suppressor gene encoding a large transmembrane protein that regulates the sonic hedgehog (SHH) signaling pathway. It acts as a negative regulator of the SHH pathway by directly inhibiting the protein smoothened (SMO) [57]. This gene plays an important role in microcephaly, developmental delay, short stature, and facial dysmorphism by stimulating the SHH pathway [58]. *PTCH1* has been reported to be associated with ear size in sheep [59].

We also identified muscle growth and fat metabolism related genes *MSTN*, *PDE3A*, *LGALS12*, *GGPS1*, and *SAR1B*. *MSTN* is a highly conserved negative regulator of skeletal muscle growth. Its deletion or mutation can lead to myocyte hyperplasia and muscle fiber hypertrophy, resulting in increased muscle mass. *MSTN* has been reported to be a major candidate gene for the double-muscle phenotype in sheep [60] and cattle [61]. *PDE3A* encodes a cyclic GMP (cGMP) and AMP (cAMP) phosphodiesterase 3A. *PDE3A* plays a prominent role in the antilipolytic action of insulin in adipocytes [62] and is reported to be involved in regulating meat production [59] in sheep. Galectin-12 (LGALS12) is an important regulator of lipid metabolism and mainly regulates prolipolytic signaling rather than promotes adipogenesis [63]. *GGPS1* gene encodes a protein with geranylgeranyl diphosphate (GGPP) synthase activity, which is an important enzyme in the isoprenoid biosynthesis pathway. Missense mutations in *GGPS1* defined a syndrome of muscular dystrophy [64,65]. Furthermore, GGPP suppresses SREBP1-dependent fatty acid biosynthesis and intracellular lipid accumulation [66], intracellular levels of neutral lipids are reduced by GGPP. *GGPS1* mutation is associated with changes in fat deposit in chicken carcasses [67]. *SAR1B* is involved in lipid metabolism in sheep [68] and is related to milk production traits in cattle [69]. In addition, we identified candidate genes related to other traits, for example, *ASIP* is associated with wool color [70], and *KRT71*, *KRT74* [71], and *IRF2BP2* [72] are involved in hair development. The keratin 74 (*KRT74*) gene and its neighboring gene *KRT71* function as genetic determinants of normal variation in hair texture across mammalian species [73] and are identified as the key genes associated with different hair types in cashmere goat and sheep [71]. *IRF2BP2* acts as a transcriptional coregulator and is also identified as a novel master gene in sheep hair development [72].

### 4.2. Selection Signatures for Tail Length Trait

Most sheep breeds bred in China are fat-tailed breeds, whose tail is used for fat deposition, and the length of the tail affects the efficiency of fat deposition. Fat-tailed sheep can be divided into long-fat-tailed breeds and short-fat-tailed breeds according to their tail length. We paid attention to screen the candidate genes associated with tail length in this study and identified candidate genes associated with tail type in long-fat-tailed breeds, including *HOXB13*, *LIN28A*, *PAX3*, and *VEGFA*.

*HOX* genes play essential roles in regulating and specifying different segment and regional identities along the developing anterior-posterior (A-P) axis of metazoans [74]. Many *Hox* genes assign identity and promote growth along the A-P axis, while *HOX13* negatively regulates embryo elongation rather than merely determining morphology, and the decrease of its activity can promote the evolution of elongated body patterns. *HOXB13*, the most 5′ gene in the *HOXB* cluster, plays a role in determining the final tail length via the repression of growth and activation of apoptosis in the caudal extremity [75,76]. *Hoxb13* mutant mice have a longer tail and premature activation of *Hoxb13* or *Hoxc13* in the axial progenitors resulting in a strong underdevelopment of tail structures [77]. The normal roles played by *Hoxb13* in patterning tails seems to be the restriction of cell proliferation and activation of programmed cell death, thus bringing a finality to the developmental program. In recent studies, *HOXB13* has been identified in sheep in China and abroad, and structural variation (insertion) of *HOXB13* has been detected to affect the tail length phenotype of sheep [9,10,78], and the insertion affects the transcription level of *HOXB13*, which may lead to the long tail phenotype in sheep. In our study, *HOXB13* showed strong selection signal in the long-tailed population via several methods. The variation of *HOXB13* may lead to the relaxation in repressing of growth. *LIN28* genes encode RNA binding proteins that function to control stem cell activity [79] and play important roles in tail bud progenitors. It has been reported that the loss of *Lin28a* in mice tail buds resulted in significant tail shortening [80,81], whereas tail bud specific overexpression of *Lin28* genes dramatically increased the caudal vertebrae number [77,80]. Further genetic studies indicated that *Lin28* genes control the activity of tail bud axial progenitors by repressing the processing of a let-7 precursor into the mature let-7 miRNA [82]. Aires et al. showed that *Lin28* and *Hox13* had opposite functions in tail bud proliferation and apoptosis, because the gene expressions of *Hox13* and *Lin28* and the corresponding phenotypic variation of tail length were negatively correlated [77]. Another study showed that the antagonistic function of *Lin28a* and *Hox13* in axial elongation may be due to the epigenetic inhibition of the *Hox13* via the Lin28a/let-7/Cbx2 pathway [81]. Therefore, *LIN28A* variations in the long-tailed population may influence downstream pathways and *HOX13* expression, breaking dynamic balance between proliferation and apoptosis in the tail bud cell. PAX3 is a transcription factor expressed in developing embryos and is a key factor in the normal formation of neurological, cardiovascular and muscular systems in mammals [83]. During myogenesis, *PAX3* is expressed in myogenic progenitor cells, regulating embryogenic myogenesis and providing a myogenic progenitor cell bank for muscle growth during development. In addition, *PAX3* plays an important role in neural development, regulating neural tube closure and neural tube modeling [84]. *PAX3* is a novel downstream gene of Wnt/β-catenin signaling, which is involved in the regulation of neural tube and tail elongation [85]. VEGFA is a key factor in the regulation of angiogenesis in adipose tissue [86]. Fat deposition is angiogenesis dependent, and the quality of adipose tissue can be regulated by the vascular system [87]. Angiogenesis of subcutaneous adipose tissue promotes the proliferation of adipose cells, thus improving the lipid storage capacity of adipose tissue, which in turn can prevent metabolic disorders of the body [88]. Therefore, the positive selection of *VEGFA* gene in the long-fat-tailed population may be associated with the level of tail fat deposition. Thus, besides *HOXB13*, *LIN28A* and *VEGFA*, *PAX3* may also be involved in the regulation of sheep tail type traits.

## 5. Conclusions

In conclusion, we characterized the genetic differences between the introduced sheep breeds and the Chinese indigenous sheep breeds, and identified novel and previously reported candidate genes by selective sweep detection. In introduced sheep populations, we identified candidate genes associated with growth rate and body size. Among them, *CRADD* is a newer selected gene in sheep, which may be associated with growth rate. *BVES*, *LIN28B*, and *WNT11* are associated with embryonic development, *HMGA2*, *MSRB3*, and *PTCH1* are associated with body size, *MSTN*, *PDE3A*, *LGALS12*, *GGPS1*, and *SAR1B* regulate muscle growth and fat deposition. *ASIP*, *KRT71*, *KRT74*, and *IRF2BP2* regulate wool color and hair development. These genes have the potential to serve as candidate genes for enhancing the growth traits of Chinese indigenous sheep. In the Chinese long-fat-tailed sheep population, tail length and fat deposition related candidate genes have been identified, among which, *HOXB13* has been reported as a major candidate gene for the long-tail phenotype of sheep. Other identified genes, *LIN28A* and *PAX3* are involved in the regulation of embryonic development, and *VEGFA* regulates angiogenesis. These genes may act as new candidate genes related to tail traits. This study will serve as a foundation for further genetic improvement of Chinese indigenous sheep and as a reference for studies related to sheep body development.

## Figures and Tables

**Table 1:** Information of the sample.

Breed	Abbr.	Sample Size	Category	Tail Type
Hu sheep	HUS	3	Chinese	Short fat tail
Sunit sheep	SNT	5	Chinese	Short fat tail
Ujimqin sheep	UJI	4	Chinese	Short fat tail
Taihang fur sheep	THF	4	Chinese	Short fat tail
Small-tailed Han sheep	STH	5	Chinese	Short fat tail
Tan sheep	TAN	5	Chinese	Long fat tail
Tong sheep	TON	3	Chinese	Long fat tail
Large-tailed Han sheep	LTH	4	Chinese	Long fat tail
Guangling Large-tailed sheep	GLT	4	Chinese	Long fat tail
Lanzhou Large-tailed sheep	LLT	2	Chinese	Long fat tail
Dorper sheep	AWD	3	Western	Thin tail
Texel sheep	TEX	5	Western	Thin tail
Hornless Poll Dorset sheep	DOP	2	Western	Thin tail
Australian Merino sheep	AME	2	Western	Thin tail
German Mutton Merino sheep	GME	3	Western	Thin tail

**Table 2:** The distribution of SNP variants in the genome region.

Catalogue	SNP Numbers
Upstream	87,385
Stop gain (Exonic)	649
Stop loss (Exonic)	178
Synonymous (Exonic)	63,547
Non-synonymous (Exonic)	75,476
Intronic	7,633,513
Splicing	3867
Downstream	117,367
Upstream/downstream	2363
Intergenic	12,638,742
ts	13,684,673
tv	7,141,784
ts/tv	1.916
Total	20,826,457

## Data Availability

The data presented in this study are openly available in Sequence Read Archive at https://www.ncbi.nlm.nih.gov/sra (accessed on 17 February 2024), BioProject ID: PRJNA521847.

## Supplementary Materials

The following supporting information can be downloaded at: https://www.mdpi.com/article/10.3390/ani14050687/s1, Table S1: The sampling location of different breeds; Table S2: List of candidate genes (*q*-value < 0.05) identified using the DCMS method for introduced sheep populations and long fat tail sheep populations.
